# Dietary exposure assessment of selected trace elements in eleven commercial fish species from the Missouri market

**DOI:** 10.1016/j.heliyon.2022.e10458

**Published:** 2022-08-30

**Authors:** Abua Ikem, Jimmie Garth

**Affiliations:** a Department of Agriculture and Environmental Sciences, Lincoln University, Jefferson City, MO 65101, United States; b Cooperative Research Programs, Lincoln University, Jefferson City, MO 65101, United States

**Keywords:** Fish species, Trace elements, Dietary intake, Human health risk, Adult population, Missouri

## Abstract

Fish is an important source of proteins, vitamins, minerals, and polyunsaturated fatty acids for nutrition adequacy. However, fish is a major link to dietary metal exposure in humans. This study describes the content of eight trace elements (As, Cd, Cr, Cu, Ni, Pb, Zn, and Hg) in eleven commercial fish species from the Missouri market and evaluated the health risks of fish muscle consumption in the adult population. Total mercury (THg) in muscle was quantified by AAS and ICP-OES was used for other elements. The recovery rates of elements from DOLT-5 reference material ranged from 83% to 106%. Of all the 239 fish samples analyzed, trace element concentrations (mg/kg wet weight) in muscle were in the following ranges: As < LOD—17.5; Cd: 0.016–0.27; Cr: 0.023–0.63; Cu: 0.034–1.06; Ni: <LOD—1.05; Pb: <LOD—0.82; Zn: 0.99–6.18; and THg: 0.0001–0.27. The levels of As, Cd, Cr, and Pb, in some samples representing several species, were above the respective limit. Kruskal-Wallis non-parametric test results showed statistically significant (p *<* 0.05) differences in Cd, As, Cr, Ni, and Hg concentrations among some pelagic and demersal species. Besides, median Hg and As levels differed (p *<* 0.05) between farmed and wild fish, with higher values observed in the wild fish samples. At times, the estimated weekly intake (EWI) for As was exceeded in certain pelagic and demersal fish. Arsenic content in some demersal fish species posed potential toxicity. Further, the incremental (ILCR) and cumulative (∑ILCR) cancer risks for As, Cr, and Ni exceeded the benchmark (10^−5^), which is a concern. Limited consumption of demersal fish species may protect adult consumers from potential health hazards.

## Highlights

• Accumulation of trace elements in eleven fish species from the market.
• As, Cd, Cr, and Pb in some species, at times, exceeded the prescribed limits.
• Elemental levels, at times, differed (p < 0.05) among pelagic and demersal species.
• Hg and As levels, higher in wild fish, differed (p < 0.05) from those of farmed fish.
• Weekly intakes of seven elements from muscle were below the respective PTWI value.
• Increased consumption per week of demersal fish species may pose health risks.

## 1 Introduction

Fish occupy a sizable portion of the human diet and positively affect global food nutrition and security (Reksten et al., 2020; Milićević et al., 2022; Chan et al., 2019). Fish is a valuable source of proteins, vitamins, minerals, microelements, polyunsaturated fatty acids (EPA: ω-3 eicosapentaenoic acid, and DHA: docosahexaenoic acid), and other nutrients essential for healthy bodily functions (Bridges et al., 2020; EFSA: European Food Safety Authority, 2014a; Jinadasa et al., 2021). The benefits of eating fish include the prevention of certain cancers, decreased mortality rates in coronary diseases, reduction in blood pressure, aiding normal neurodevelopment in children, gut microbiota modulation, skin protection, and others (Anual et al., 2018; Chen et al., 2022; Morales and Higuchi, 2018). Despite the health benefits of fish consumption, there is a worldwide public concern about human exposure to chemical contaminants (Varol and Sünbül, 2020; Burger and Gochfeld, 2005; Bridges et al., 2020).

Trace elements in aquatic systems may arise from natural (e.g., release from rocks, soil, and sediment; volcanoes) and anthropogenic (e.g., runoffs, industry and agricultural wastewater discharges, waste incineration, atmospheric deposition) sources (Kolarova and Napiórkowski, 2021). For decades, the global anthropogenic emissions of trace elements are comparable to or even larger than natural emissions (Pacyna and Pacyna, 2001). Metals such as Cu, Cr, Ni, and Zn are essential and involved in several biochemical reactions for normal human physiological function (WHO, 1996). In contrast, nonessential elements such as Hg, As, Cd, and Pb are recognized priority pollutants with no nutritional benefits according to the United States Environmental Protection Agency (US EPA; US EPA, 2014). The highlighted priority pollutants are toxic, non-degradable in the environment, and have bioaccumulation capacity in the food chain. The significant symptoms of metal toxicities in humans include intelligence quotient decrements, especially in children, various cancers, macromolecules (e.g., DNA, proteins) and bone damage, kidney and liver disorders, endocrine and reproductive effects, and so forth (Gupta et al., 2019; ATSDR, 2021).

Previous studies found contaminants such as Pb, Hg, As, Cd, CH_3_Hg (methylmercury), and other organic compounds in fish (McGoldrick and Murphy, 2016; Cunningham et al., 2019; Cáceres-Saez et al., 2018; Pico et al., 2019). Fish are exposed to heavy metals from feeds and wild sources. The degree of exposure of fish to contaminants may depend on the metal concentration, age, size, foraging depth (Alam et al., 2002), exposure duration, reproductive cycle, and environmental factors (e.g., temperature differences, salinity, pH changes, etc.).

About 71% of seafood supplied to the United States (U.S.) is imported from Asia (Love et al., 2021) while other supplies are from the wild and aquaculture (National Oceanic and Atmospheric Administration, 2019). Cultured fish may bioaccumulate metals due to their feeding habits, feed quality, age, size, and other factors (Maule et al., 2007). Fish feeds, at times, contain metals above the prescribed limits (Mannzhi et al., 2021; Maule et al., 2007). Other studies found chemical contaminants such as PCBs (McMullin et al., 2022) and total Hg (Ikem et al., 2021a) in feeds. Consequently, seafood may contribute to the total dietary intake of chemical contaminants in humans. Consumers in the U.S. consider imported seafood as less safe relative to domestic products (Love et al., 2021; Siegner, 2013), and food inspection by U.S. Federal agencies is at the lowest level (Love et al., 2021). Also, trust among consumers might be lower for fish imported from a developing country (Wang et al., 2013). Besides, the use of unsafe chemicals in aquaculture abroad (Li et al., 2022) and the few regulations (Done and Halden, 2015) are a public concern.

The assessment of human health risks from toxic metals through fish consumption (Milićević et al., 2022; Olmedo et al., 2013; Kollander et al., 2019) has global interest due to the propensity of fish to uptake metals from their environment. Bioaccumulation of contaminants in wild fish varies across species and geographical areas (Ho et al., 2021). In the case of farm-raised fish, there might be a variation in the quality of feeds, processes, and regulations governing aquaculture operations. Hence, appraisal of risks is important in the management of disease in the population, provision of health-based information for consumers, and the improvement of food quality. Exposure risk evaluation may follow the deterministic and probabilistic approaches (Meerpool et al., 2021). Factors such as food intake rate, the weight of the individual, analyte concentration, reference dose, and cancer slope factors are applied in the estimation of risks.

This study determined the concentrations of eight selected trace elements (As, Cd, Cr, Cu, Ni, Pb, Hg, and Zn) in eleven commercial fish species from the Missouri retail market and compared the values with maximum limits. Additionally, the present work evaluated the dietary exposure risks from metals/metalloid via fish muscle consumption in the adult class. To the best of our knowledge, this is the first comprehensive study on dietary exposure risks through the consumption of various fish species from the Missouri market. The goal of this study was to provide a baseline dataset, enhance an understanding of the potential dietary exposure risks, and support future consumption advisories.

## 2 Materials and methods

### 2.1 Chemicals, reagents, and gases

Ultrapure water (18.2 MΩ cm^−1^ at 25 °C) was produced by a Milli-Q® Integral 5 water purification system (Millipore Corporation, Massachusetts, USA). All glassware and polyethylene bottles were acid-cleaned (30% v/v HNO_3_ for 24 h) and thoroughly rinsed with ultrapure water followed by drying at room temperature. Concentrated nitric acid (HNO_3_; 65%, w/w; trace metal-grade), mercury (Hg; 1000 mg L^−1^), and yttrium (Y; 1000 mg L^−1^) stock standards were procured from Fisher Scientific (IL, USA). Multi-element calibration standard solution (100 mg L^−1^) was acquired from SPEX Certiprep (NJ, USA). Agilent Technologies (Santa Clara, CA, USA) supplied the tune stock solution for ICP internal calibration. The independent calibration verification (ICV) solution and quality control sample (QCS-26) were provided by High Purity Standards (Charleston, SC, USA). SRM 1640a (trace elements in water) from the National Institute of Standards and Technology (Gaithersburg, MD, USA), and DOLT-5 (dogfish liver certified reference materials for trace metals and other constituents) from the National Research Council (Ottawa, ON, Canada) were procured for validation and accuracy measurements. Nitrogen and argon gases (99.995% purity) were delivered by Airgas Mid-America (Holts Summit, MO, USA). Nitrogen gas was used in the pressurization of the microwave digester while argon gas was utilized in the generation of the plasma and sample aspiration during ICP analysis.

### 2.2 Sample collection

A total of 239 fish muscle samples that included wild-caught (n = 180), and cultured (n = 59) species were randomly purchased from the Missouri market between February and May 2019. The samples represented both pelagic and demersal species due to their habitats, trophic levels, and feeding habits. The eleven species sampled were the most commonly available fillet or frozen fish retailed by the major supermarkets and outlets. The fish samples were Alaskan Pacific cod (*Gadus macrocephalus*, USA, n = 21); Alaskan sockeye salmon (*Oncorhynchus nerka*, USA, n = 20); ahi tuna—yellowfin (*Thunnus albacares*, Vietnam, n = 12); tilapia (*Oreochromis niloticus*, China, n = 22); Alaska pollock (*Gadus chalcogrammus*, USA, n = 15); channel catfish (*Ictalurus punctatus*, USA, n = 25); Atlantic salmon (*Salmo salar*, Chile, n = 12); pink salmon (*Oncorhynchus gorbuscha,* China, n = 20); Pacific cod (*Gadus microcephalus*, China*;* n = 18); North Atlantic ocean perch (*Sebastes Norvegicus*, USA, n = 18); olive flounder (*Paralichthys olivaceus*, China, n = 16); Pacific whiting (*Merluccius productus*, USA, n = 20); and ahi tuna (*Thunnus albacares,* Indonesia, n = 20). Table 1 presents the common and scientific names, the origin of fish, and the product label of the commercial fish species from the Missouri market. All frozen samples were placed in polyethylene bags and transported to the laboratory in coolers containing ice. Samples were then coded for easy identification and frozen in an ultralow freezer at −40 °C until chemical analyses.

**Table 1:** Common and scientific names, habitat, origin, and product label of commercial fish species from the Missouri market.

Scientific name	Common name	Family	Habitat^a^	Origin	n^b^	Wild or farmed	Product label
*Oncorhynchus nerka*	Alaskan sockeye salmon	Salmonidae	Pelagic	USA	20	W	All-natural; skin-on-fillet
*Salmo salar*	Atlantic salmon	Salmonidae	Pelagic	Chile	12	F	Skinless; boneless fillet; skinless; boneless fillet; artificial color added
*Oncorhynchus gorbuscha*	Pink salmon	Salmonidae	Pelagic	China^c^	20	W	Skin-on; boneless fillet
*Thunnus albacares*	Ahi (Yellowfin) tuna	Scombridae	Pelagic	Vietnam	12	W	Filtered wood smoke used to preserve color
*Thunnus albacares*	Ahi (Yellowfin) tuna	Scombridae	Pelagic	Indonesia	20	W	Skinless; filtered wood smoke used to preserve color
*Oreochromis niloticus*	Tilapia	Cichlidae	Pelagic	China	22	F	Skinless; boneless fillet
*Gadus chalcogrammus*	Alaska pollock	Gadidae	Benthopelagic	USA	15	W	Skinless; boneless fillet
*Ictalurus punctatus*	Channel catfish	Ictaluridae	Benthic	USA	25	F	Skinless; boneless fillet
*Sebastes Norvegicus*	North Atlantic Ocean perch	Sebastidae	Pelagic	USA	18	W	Skin-on fillets; sodium citrate and ascorbic acid to maintain color, salt as a preservative
*Paralichthys olivaceus*	Olive flounder	Paralichthyidae	Benthic	China	16	W	Boneless and skinless fillet; sodium tripolyphosphate (to retain moisture)
*Gadus macrocephalus*	Alaskan Pacific cod	Gadidae	Benthic	China	18	W	Boneless; skinless fillet; sodium tripolyphosphate
*Gadus macrocephalus*	Alaskan Pacific cod	Gadidae	Benthic	USA	21	W	All-natural; skinless fillet; Skinless; boneless fillet
*Merluccius productus*	Pacific whiting	Merlucciidae	Pelagic	USA	20	W	Skin-on fillet

a Habitat description from www.fishbase.org.

b n is the number of samples analyzed.

c Wild-caught in Russia. W = Wild; F = farmed.

### 2.3 Mineralization of fish muscle samples

Microwave digestion of fish muscle tissue samples without skin was performed in a single reaction chamber (SRC) UltraWAVE™ digestion system (Milestone Inc., CT, USA) with the capacity to reach the maximum pressure of ∼200 bars, and a maximum temperature of 300 °C. Approximately 0.3 ± 0.10 g (wet weight (ww)) of fish muscle was accurately weighed into an acid-cleaned quartz digestion vessel followed by the addition of an aliquot (4 ml) of concentrated nitric acid. For method validation, DOLT-5 standard reference material was digested along with samples. Additionally, blanks were analyzed during each digestion batch to check for contamination. The digestion of each fish muscle sample followed the six-step microwave heating program as follows: (i) 1500 W: ramp 5 min to 70 °C (gentle breakup of the sample); (ii) 1500 W: ramp 5 min to 100 °C (gentle breakup of the sample); (iii)) 1500 W: ramp 5 min to 180 °C (bond breakage and mineralization); (iv) 1500 W: ramp 10 min to 250 °C (complete mineralization of sample); (v) 1500 W: hold at 250 °C (complete mineralization of sample), and (vi) cooling of digest and depressurization of the SRC. Each cooled digest was quantitatively transferred into an acid-cleaned 50 ml standard flask and made up to volume with ultrapure water. Each fish muscle sample was digested in triplicate.

### 2.4 Analyses of trace elements in fish muscle

#### 2.4.1 Determination of trace elements in fish muscle using ICP-OES and quality assurance

The concentrations of trace elements (Cr, Cu, Ni, Zn, As, Cd, and Pb) in digested fish muscle samples were determined using the Agilent 5110 synchronous vertical dual view inductively coupled plasma—optical emission spectrometer (ICP-OES; Agilent Technologies, California, USA). The analysis complied with the international guideline, EN ISO/IEC 17025:2017 (ISO, 2017). The instrument conditions and operational settings were previously described (Ikem et al., 2021b). The tuning process, calibration, validation, and analysis of samples were as recommended by Agilent. The wavelengths (nm) of measurements were Cd: 214.439; As: 188.980; Cu: 327.395; Ni: 231.604; Cr: 267.716; Pb: 220.353; and Zn: 213.857. Elemental analysis of samples was performed under the axial view mode and the ICP equipment was optimized daily. The Agilent ICP Expert software (Version 7.4.1. 10449) controlled the equipment, autosampler, plotted the calibration graphs, and provided the elemental concentrations.

The limit of detection (LOD), the limit of quantitation (LOQ), trueness, and precision followed the EURACHEM criteria (EURACHEM, 2014). The LOD and LOQ values were calculated as three times the standard deviation (3.3 s) and ten times the standard deviation (10 s), respectively (EURACHEM, 2014) of results from the analysis of twenty spiked (5 μg/l) blanks. Table 2 shows the LOD values (μg/l) for the trace elements and the recoveries of metals/metalloid from the DOLT-5 reference (ISO 5725-2 guide: ISO, 2019). The LODs (μg/l) and LOQs (μg/kg) in parenthesis, for the elements were As: 9.5 (29); Cd: 5.0 (16); Cr: 6.0 (17); Cu: 1.0 (3.0); Ni: 3.0 (9.0); Pb: 5.0 (16); Zn: 3.0 (8.0); and Hg: 0.0002 (0.0006). Quality control measures performed during the experiments included the cleaning of all glassware with 30% nitric acid followed by a thorough rinse with ultrapure deionized water, appropriate preparation of working standards, and analysis of blanks, ICV solution, and internal standard (Y), and other standards (SRM 1640a, DOLT-5, and QCS-26). Moreover, recalibration of the instrument was performed after every ten sample runs in a sequence. The coefficients of determination (*R*^2^) for the ICP—OES calibration of the seven elements were greater than 0.995.

**Table 2:** Limit of detection (LOD^a^^,^^b^; μg/L), limit of quantitation (LOQ^a^^,^^b^; μg/kg) and the recovery values of trace elements from DOLT-5 (n = 7; mg/kg) by ICP—OES^c^ (n = 7) and AAS^d^ (n = 5).

Element	Λ (nm)	LOD	LOQ	DOLT- 5: CV	DOLT-5: FV	% Rec.
As	188.980	9.5	29	34.6 ± 2.4	28.8 ± 0.8	83
Cd	214.439	5.0	16	14.5 ± 0.6	13.4 ± 0.03	92
Cr	267.716	6.0^b^	17^b^	2.35 ± 0.58	2.50 ± 0.20	106
Cu	327.395	1.0	3.0	35.0 ± 2.4	36.1 ± 0.11	103
Ni	231.604	3.0	9.0	1.71 ± 0.56	1.61 ± 0.16	94
Pb	220.353	5.0	16	0.162 ± 0.032	0.13 ± 0.04	80
Zn	213.857	3.0	8.0	105.3 ± 5.4	90.1 ± 0.50	86
Hg^d^	253.65	0.0002^d^	0.0006	0.44 ± 0.18	0.43 ± 0.3	98

CV = Certified value; FV = Found value

a LOD of trace elements using ICP—OES was calculated from analysis of 20 blanks.

b Estimated from 20 runs of a 5 μg/l spiked solution; Rec. % = recovery percentage.

c All ICP—OES measurements were performed under the axial view mode.

d Hg analysis was by AAS (atomic absorption spectrometry; ^d^ LOD and LOQ were estimated from the analysis of pre-cleaned boats subjected to the analytical cycle).

The accuracy (%) result from the analysis of DOLT-5 certified reference was calculated following Eq. (1):

(1) $$A\;=100\;\times \frac{C}{R}\;$$

where A is the accuracy rate (%) for the trace element, C is the measured element concentration in fish muscle, and R is the certified value provided for the element.

Elemental concentrations in fish muscle, expressed as mg/kg ww, were calculated from Eq. (2):

(2) EC = [(AC/W) × V × DF]

where EC is the element concentration in fish muscle (μg/g ww), AC = analytical concentration result (μg/ml), W = fish muscle weight (g), V = volume of digested sample (ml), and DF = dilution factor.

The analysis of QCS-26 and ICV solutions produced satisfactory recovery values ranging from 98% to 101% for the analyzed elements. Also, the accuracy rates from the analysis of SRM 1640a and DOLT-5 reference samples were in the acceptance range (83%–106%) per the ISO 5725-2 guide (ISO, 2019). The recoveries from SRM 1640a and DOLT-5 ranged from 97%–102% and 83%–106%, respectively, and the relative standard deviations (RSDs) ranged from 1% to 3%.

#### 2.4.2 Mercury analysis of fish muscle by AAS (Direct Mercury Analyzer: DMA-80 Evo)

Determination of total Hg (THg) concentrations in fish muscle samples was performed using a Mercury Auto Analyzer (DMA-80 Evo –TRICELL; Direct Mercury Analyzer, Milestone, Inc., USA) per the US EPA method 7473 (US EPA, 2007). The analytical method followed the operational sequence: thermal decomposition of the sample, catalytic conversion, amalgamation, and mercury detection by atomic absorption spectrophotometry at 253.65 nm. Samples were accurately weighed into cleaned quartz boats and subjected to the analytical cycle settings as follows: drying temperature/time (90 s–200 °C); decomposition ramp (120 s–650 °C); decomposition hold (90 s–650 °C); catalyst (565 °C); purge time (60 s); and amalgamation time (12 s at 900 °C); recording time (30 s), and ultrapure oxygen (99.99% purity; flow: 120 ml/min). The EasyControl software controlled the equipment operation. Calibration working solutions were prepared through serial dilutions from a 1000 ppm Hg standard. Cell 0 was calibrated with 0.5, 1, 1.5, and 2 Hg amounts (ng) prepared from a 0.01 mg Hg/L solution; Cell 1: was calibrated with 3, 5, 10, 15, and 25 Hg amounts (ng) prepared from a 0.1 mg Hg/L; and Cell 2: was calibrated with 30, 50, 100, 200 and 300 Hg amounts (ng) prepared from a 1.0 mg Hg/L. The fitted instrument calibration curve for the analysis of batch samples produced a coefficient of determination (*R*^2^) value greater than 0.996. Absorbance at a wavelength of 253.65 nm was measured as a function of the concentration in each sample. The ˋconcentratioń procedure was applied when the mercury content in a sample was below detection in a single sample run. Blank readings were generally <0.0001 ng Hg. The accuracy of the method was verified from the analysis of the DOLT-5 reference. Each fish muscle sample was analyzed for THg in triplicate.

### 2.5 Human health risk evaluation

#### 2.5.1 Comparison of metals/metalloid levels in fish muscle with thresholds

Metals/metalloid concentrations found in the fish samples (this study) were compared to the permissible limits for fish (FAO, 1983; Official Journal of the European Union, 2008, Official Journal of the European Union, 2014, Official Journal of the European Union, 2015; Ministry of Agriculture, Forestry and Fishery, United Kingdom: MAFF, 1998; Canadian Food Inspection Agency: CFIA, 2019; ABIA, 1998; and Egyptian Organization for Standardization: EOS, 1993) to assess the potential risks to consumers.

#### 2.5.2 Estimated daily/weekly intakes (EDI/EWI)

Estimated daily/weekly intakes (EDI/EWI) are dependent on metals/metalloid concentrations, the amount of food consumed per day, and the individual's body weight. The total amount of an ingested contaminant may not reflect the amount available to the human body (Maisanaba et al., 2017). Consequently, total metal concentrations from fish muscle consumption in the present work may not be 100% bioavailable. The dietary risk in consumers is dependent on biochemical factors (e.g., rate of assimilation and elimination of metals). This study applied total metal concentrations in the estimation of dietary risk in adults.

The risks to human health from the consumption of fish species expressed as daily exposure (Varol and Sünbül, 2020) followed Eq. (3):

(3) EDI=[EC×IR]/BW

where EDI is the estimated daily intake (μg/kg body weight per day), EC is the average element concentration in fish muscle (μg/g ww), IR is the ingestion rate (amount of fish consumed in one day; 32.5714 g/person/day assumed; Ikem and Egilla, 2008), and BW is the average body weight (70 kg assumed for the adult population in the United States; US EPA, 1989).

The EWI expressed as the weekly exposure was calculated according to Eq. (4):

(4) EWI=EDI×F

where EWI is the estimated weekly intake (μg/kg body weight per week) assuming 70 kg body weight for the United States adult population, EDI parameters were previously described, and F is the number of days in a week fish is consumed (7 days assumed in this study). The EWI values were compared with the US EPA oral reference dose (RfD_o_; US EPA, 2019) and other limits (EFSA, 2009a, 2012, 2014b; WHO, 1993; JECFA, 2000; ATSDR, 2007).

#### 2.5.3 Estimation of non-cancer and cancer risks

Health indices (THQ: target hazard quotient; TTHQ: total target hazard quotient; ILCR: incremental lifetime cancer risk; and ΣILCR: cumulative incremental lifetime cancer risk) for metals/metalloid through fish consumption were estimated for the adult population. The calculated values were compared to a cancer risk benchmark (10^−5^; US EPA, 2000; US EPA, 1989; US EPA, 1991) to assess exposure risks. THQ is an indicator of risk, expressed as the ratio between exposure and the RfD_o_ or provisional tolerable daily intake (PTDI) for the element. A ratio that is greater than one (i.e., THQ >1) implies that the exposed population may be at risk (US EPA, 2000). Conversely, a THQ value less than 1 or equal to 1.0 (i.e., THQ ≤1), indicates no adverse effect from the consumption of fish. The estimation of THQ values followed Eq. (5):

(5) $$THQ=\frac{\left[\left(EDI\right)\left(EFr\;\times \;ED\right)\right]}{\left[\left(RfD_{o}\;\times \;AET\right)\right]}\;\times \;10^{-3}$$

where THQ is the target hazard quotient, EDI is the estimated daily intake (μg/kg body weight per day; EDI parameters were presented earlier), EFr is the exposure frequency (365 days/year), ED is the exposure duration (79 years assumed as the average lifetime for the United States population according to the Center for Disease Control and Prevention: CDC; https://www.cdc.gov/nchs/data/hus/2019/004–508.pdf; Center for Disease Control and Prevention (CDC), 2021), AET is the averaging exposure time (365 days/year × 79 years = 28,835 days), and RfD_o_ is the oral reference dose (mg/kg body weight per day) for inorganic As (iAs; the most toxic form): 3.0 × 10^−4^; Cd: 1.0 × 10^−3^; Cu: 4.0 × 10^−2^; Cr (as Cr (VI)): 3.0 × 10^−3^; Pb: 3.6 × 10^−2^; Ni subsulfide: 1.1 × 10^−2^; Hg: 1.0 × 10^−4^; and Zn: 3.0 × 10^−1^ (US EPA, 2020; US EPA, 2019).

Exposure to more than one contaminant from fish muscle consumption may be associated with combined or interactive effects (Li et al., 2013). Hence, the sum of more than one hazard quotient for multiple substances (US EPA, 1989) expressed as TTHQ followed Eq. (6):

(6) TTHQ_Fish muscle_ = THQ_(Zn)_ + THQ_(Pb)_ + --- + THQ_(As)_ + THQ_(Ni)_

TTHQ ≤1.0 value implies that insignificant adverse effects are predicted and if TTHQ >1.0, then chronic toxic effects are probable (US EPA, 1989).

The ILCR describes the incremental probability that an individual will develop cancer during one's lifetime from specific exposure to a carcinogenic compound (US EPA, 2001). In the current work, the ILCR and ΣILCR through the consumption of fish muscle were compared to the cancer benchmark (10^−5^). The ILRC was estimated using the daily intakes (this study) and the cancer slope factors (CSF; mg/kg per day) for the trace elements according to Eq. (7):

(7) ILCR=CDI×CSF

where CSF is the cancer slope factor (a plausible upper-bound estimate of the probability of a response per unit intake of a chemical over a lifetime) (US EPA, 1989). The CSF (mg/kg per day) for As (inorganic arsenic), Cr (VI), Ni (nickel subsulfide), and Pb (subacetate) used in the calculation were 1.5, 0.5, 1.7, and 0.0085, respectively (US EPA, 2019). The CSF values for other analyzed elements were not furnished by the US EPA.

CDI (mg/kg/day), which is the chronic daily intake of a chemical (i.e., the average daily dose of exposure from a chemical; US EPA, 1989), was estimated according to Eq. (8):

(8) $$CDI=\frac{\left[EDI\;\times \;EFr\;\times ED)\right]\;}{AET\;}\times \;10^{-3}$$

where EDI is the estimated daily intake (μg/kg body weight per day; EDI parameters were described earlier), EFr is the exposure frequency (365 days/year), ED is the exposure duration (79 years; average lifetime exposure in the United States), and AET is the averaging exposure time (365 days/year × 79 years = 28,835 days).

Acceptable cancer risk levels for carcinogenic chemicals range from 1 × 10^−6^ (i.e., the risk of developing cancer is 1 in 1,000 000) to 1 × 10^−4^ (i.e., the risk of developing cancer is 1 in 10, 000) (US EPA, 2001). Therefore, an ILCR <10^−6^ implies negligible cancer risk while ILCR >10^−4^ signifies potential cancer risk (US EPA, 1991) from metal exposure via fish consumption. This study applied an acceptable cancer risk benchmark of 10^−5^ (i.e., the risk of developing cancer is 1 in 100,000). Besides, the cumulative cancer risk (∑ILCR) from exposure to four trace elements (As, Cr, Ni, and Pb) from fish muscle (this study) was estimated from the individual metal/metalloid incremental risks.

### 2.6 Statistical analyses

Triplicate results from ICP analysis were averaged and grouped according to fish species. The concentrations of trace elements in fish muscle were calculated on a wet weight basis. Descriptive statistics of the experimental results expressed as mean ± standard deviation was prepared using Microsoft™ Excel software (Microsoft Office Professional Plus, 2016; Microsoft Corporation, USA). Normality and homogeneity of variances in the dataset were checked using Shapiro-Wilk W (Statgraphics Centurion 18-X64 version 17.1.04; Statpoint Technologies, USA). Also, a multivariate normality test following Royston's test comparison with a Chi-square distribution suggested a non-normal multivariate distribution of the dataset. Thus, the non-parametric Kruskal-Wallis test (Statgraphics Centurion 18 × 64) was applied to ascertain the differences in metal/metalloid concentrations across the fish species. Further, Spearman's correlation was performed to evaluate the interrelationships of the analyzed trace elements with one another. Statistical significance was accepted when p ≤ 0.05.

## 3 Results and discussion

### 3.1 Quality assurance results

The ICV (1 mg/L), QCS-26 (0.5 mg/L), and SRM 1640a analysis results were within the acceptance criteria, with recoveries (%) in the ranges from 97–99.7, 97.9–101, and 83–112, respectively. Regarding elemental analysis by ICP, the accuracy results from the analysis of DOLT-5 gave satisfactory recovery rates ranging from 83% to 106%. Concerning mercury analysis, the accuracy rate from the DOLT-5 reference was 98% (Table 2), which was within the acceptance range with the RSDs, in the range from 1% to 2%.

### 3.2 Metals/metalloid concentrations in fish species

A total of 239 fish muscle samples belonging to eleven commercial fish species were purchased from the Missouri market and analyzed for eight selected trace elements (As, Cd, Cr, Cu, Ni, Pb, Hg, and Zn). The fish species analyzed were either wild-caught or farm-raised (Table 1). Of all the species examined in this work, tilapia, Atlantic salmon, and catfish species were farmed. All the fish species investigated in this study were Alaskan Pacific cod: USA; Alaskan sockeye salmon; ahi tuna: yellowfin; tilapia; Alaska/walleye pollock; channel catfish; Atlantic salmon; pink salmon; Pacific cod: China; North Atlantic Ocean perch; olive flounder; Pacific whiting; and ahi tuna–Indonesia.

Table 3 shows the statistical summary values (average ± standard deviation) of eight selected trace elements in fish species samples from the Missouri market. For comparison, Table 4 summarizes the literature values on metals/metalloid in fish species. Except for As and Cd, detectable levels of other analyzed trace elements were found in fish muscle samples examined. Of all analyzed fish samples (n = 239) in this study, the range of trace element concentrations (mg/kg ww) were Cr: 0.023–0.63; Ni: <LOD—1.05; As: <LOD—17.5; Cd: 0.016–0.27; Cu: 0.034–1.06; Pb: <LOD—0.82; Zn: 0.99–6.18; Hg: 0.0001–0.27. There was a wider variability of As concentrations in Pacific cod samples, a high-level predator fish, in comparison with the levels in other sampled species. The abundance of the essential elements in fish muscle was in the order: Zn > Cu > Cr > Ni; while the potentially toxic elements (PTEs) followed the trend: As > Pb > Hg > Cd.

**Table 3:** Average (± standard deviation) concentrations (mg/kg wet weight) of trace elements in the muscle tissue of fish species from the Missouri market.

Fish species and number of samples	Origin	As	Cd	Cr	Cu	Ni		Pb	Zn	Hg
*Pelagic species*										
Alaskan Sockeye salmon (n = 20)	USA	0.14 ± 0.10	0.038 ± 0.027	0.13 ± 0.12	0.40 ± 0.20	0.14 ± 0.11		0.18 ± 0.09	2.89 ± 0.86	0.043 ± 0.011
Atlantic salmon (n = 12)	Chile	0.05 ± 0.05	0.026 ± 0.012	0.13 ± 0.09	0.24 ± 0.04	0.09 ± 0.08		0.14 ± 0.13	2.89 ± 0.33	0.005 ± 0.003
Pink salmon (n = 20)	China	0.19 ± 0.11	0.022 ± 0.004	0.14 ± 0.04	0.42 ± 0.13	0.06 ± 0.04		0.15 ± 0.08	3.24 ± 0.64	0.019 ± 0.004
Ahi tuna (n = 12)	Vietnam	1.43 ± 0.18	0.079 ± 0.010	0.17 ± 0.03	0.33 ± 0.06	0.12 ± 0.02		0.21 ± 0.06	3.62 ± 0.83	0.073 ± 0.054
Ahi tuna (n = 20)	Indonesia	0.54 ± 0.26	0.036 ± 0.058	0.11 ± 0.11	0.27 ± 0.12	0.10 ± 0.10		0.19 ± 0.15	2.91 ± 0.87	0.10 ± 0.073
Tilapia (n = 22)	China	0.05 ± 0.07	0.065 ± 0.035	0.21 ± 0.07	0.27 ± 0.10	0.13 ± 0.05		0.18 ± 0.08	2.84 ± 0.58	0.001 ± 0.001
Ocean perch (n = 18)	USA	0.47 ± 0.18	0.018 ± 0.002	0.09 ± 0.08	0.14 ± 0.09	0.09 ± 0.12		0.13 ± 0.09	2.38 ± 0.27	0.039 ± 0.035
Pacific whiting (n = 20)	USA	0.18 ± 0.15	0.020 ± 0.003	0.14 ± 0.16	0.30 ± 0.19	0.15 ± 0.25		0.17 ± 0.08	2.58 ± 0.29	0.048 ± 0.049
*Benthic species (demersal)*										
Pollock (n = 15)	USA	1.22 ± 0.28	0.020 ± 0.001	0.11 ± 0.04	0.25 ± 0.13	0.07 ± 0.04	0.22 ± 0.17		2.53 ± 0.56	0.009 ± 0.003
Catfish (n = 25)	USA	0.08 ± 0.08	0.027 ± 0.016	0.12 ± 0.06	0.17 ± 0.11	0.07 ± 0.08	0.16 ± 0.07		2.72 ± 1.03	0.003 ± 0.002
Flounder (n = 16)	China	2.13 ± 0.52	0.020 ± 0.002	0.16 ± 0.05	0.24 ± 0.05	0.07 ± 0.04	0.17 ± 0.07		3.70 ± 0.69	0.040 ± 0.016
Pacific cod (n = 18)	China	2.15 ± 0.94	0.033 ± 0.036	0.17 ± 0.04	0.15 ± 0.06	0.09 ± 0.05	0.17 ± 0.10		2.56 ± 0.49	0.040 ± 0.022
Pacific cod (n = 21)	USA	3.89 ± 3.98	0.038 ± 0.030	0.11 ± 0.08	0.17 ± 0.10	0.10 ± 0.04	0.16 ± 0.09		2.34 ± 0.92	0.063 ± 0.037
All countries (This study, mg/kg)	Average for all fish species (n = 239)	0.94	0.033	0.14	0.30	0.10	0.17		2.83	0.036
	Median	0.31	0.020	0.12	0.24	0.08	0.16		2.74	0.025
	Minimum	<LOD	0.016	0.023	0.034	<LOD	<LOD		0.99	0.0001
	Maximum	17.54	0.27	0.63	1.06	1.05	0.82		6.18	0.272^i^
	P95	3.01	0.09	0.27	0.50	0.26	0.32		4..04	0.12
MAFF, 1998 (mg/kg)			0.2		20		2.0		50	0.5
Official Journal of the European Union, 2008, Official Journal of the European Union, 2014, Official Journal of the European Union, 2015 (mg/kg)			0.10^a^; 0.05^b^				0.3^c^			1.0^d^
Other limits (mg/kg)		3.5^e^		0.10^f^	30^g^	10^h^			30^g^	

SD: Standard deviation; P95: 95% percentile values; LOD in μg/kg LOD in μg/kg (Cd: 5; Ni: 3; As: 9.5; Cr: 6.0; Cu: 1.0; Pb: 5; Hg: 0.0002; and Zn: 3.0); MAFF, 1998 (Ministry of Agriculture, Forestry and Fisheries).

^a, b^Official Journal of the European Union, 2008, Official Journal of the European Union, 2014, Official Journal of the European Union, 2015; ^a, b^ Maximum Cd level for certain fish species, e.g., tuna, sardine, mackerel, etc.

c Official Journal of theEuropean Union, 2015 (Muscle meat of fish).

d Official Journal of theEuropean Union, 2008; Maximum Hg level permitted in tuna (*Thunnus species*).

e CFIA, 2019 (Canadian Food Inspection Agency).

f ABIA (Associação Brasileira das Indústrias da Alimentação), 1998.

g FAO (Food and Agriculture Organization), 1983.

h EOS (Egyptian Organization for Standardization), 1993.

**Table 4:** Heavy metals accumulation in fish species (this study) in comparison with literature values.

Fish species	As	Hg	Ni	Cd	Cr	Cu	Zn	Pb	References
All samples^a^^,^^b^ (eleven species, Missouri, USA)	0.94	0.036	0.10	0.033	0.14	0.30	2.83	0.17	This study
Flounder a^*,*^^b^ (New Jersey, USA)	3.3	0.05	NA	0.01	0.31	NA	NA	0.06	Burger and Gochfeld (2005)
Pink Salmon a^*,*^^b^ (*Oncorhynchus gorbuscha;* Alaska, USA)	0.212	0.0419	NA	0.0027	NA	NA	NA	0.027	Burger et al. (2014)
Tuna^a^∗	1.43	NA	NA	NA	NA	NA	NA	NA	Ruttens et al. (2012)
Whiting^a^^,^∗ (Belgium)	5.37	NA	NA	NA	NA	NA	NA	NA	Ruttens et al. (2012)
Catfish a^*,*^^b^ (*Clarias fuscus*, China)	0.04	NA	2.58	0.02	0.54	1.40	27.8	0.37	Leung et al. (2014)
Atlantic bluefin tuna a^*,*^^b^ (*Thunnus thynnus*, Spain)	NA	0.52		0.02	NA	NA	NA	0.30	Milenkovic et al. (2019)
Tub Gurnard a^*,*^^b^*(Triglia lucerne*; İskenderun Bay, Turkey)	1.38	NA	0.72	0.01	0.65	4.19	28.2	0.14	Yilmaz et al. (2010)
Atlantic cod^a^^b^ (*Gadus morhua L.;* Baltic Sea, Poland)	0.13–7.6	0.019–0.646	NA	0.002–0.008	0.02–0.06	0.08–0.48	2.4–5.5	0.003–0.043	Polak-Juszczak and Podolska (2021)
Catfish^a^^*,*^^b^ (*Hypostomus sp.;* Brazil)	NA	0.07	NA	NA	NA	NA	NA	NA	Custódio et al. (2020)
Tuna^a^^*,*^^b^ (*Thunnus sp;* Brazil)	NA	0.08–0.61	NA	NA	NA	NA	NA	NA	Custódio et al. (2020)
Nile tilapia^a^^,^^c^ (*Oreochromis niloticus;* Brazil)	NA	nd—0.09	NA	NA	NA	NA	NA	NA	Custódio et al. (2020)
*Balistes capriscus*^a, b^	NA	NA	0.09	0.08	0.27	0.85	4.18	0.53	Lozano-Bilbao et al. (2021)
*Canthidermis sufflamen*^a, b^	NA	NA	0.51	0.03	0.14	1.23	7.35	0.37	Lozano-Bilbao et al. (2021)
*Heteropriacanthus fulgens^a, b^*	NA	NA	0.08	0.02	0.31	1.24	5.47	0.40	Lozano-Bilbao et al. (2021)

nd = not detectable; NA = not analyzed.

a Values are ranges or averages expressed as mg/kg wet weight.

b Wild fish analyzed.

c Farmed fish analyzed.

∗ Wild or farmed not indicated.

The metalloid, arsenic (As) occurs in several forms, of which inorganic arsenic is the most toxic form. Inorganic As (iAs, i.e., sum of As^III^ and As^V^) form is carcinogenic and food is the major source of As exposure in the population. Arsenic (As) is a non-essential element and not required in animal metabolism. The forms of As in foods include arsenate, dimethylarsinate, arsenobetaine, arsenosugars, and others (EFSA, 2009b). Almost 4.2% of all samples exceeded the As limit (3.5 ppm). Among all species, the highest average As concentration (3.92 mg/kg) was observed in demersal fish (Pacific cod–USA) while the lowest mean level (0.05 mg/kg) was found in two pelagic fish samples (tilapia and Atlantic salmon). Other species with average values (mg/kg) above 1.0 mg/kg were Pacific cod (China, 2.15); flounder (2.13); ahi tuna: Vietnam (1.43); and pollock (1.22). Approximately 13% of samples contained As above 2 mg/kg. Acute high-dose oral exposure to iAs may cause nausea, vomiting, diarrhea, cardiovascular effects, and encephalopathy (ATSDR, 2007). The mean As concentration in Pacific cod–USA (3.92 mg/kg) in the present study exceeded the Canadian prescribed limit (3.5 mg/kg) for fish (CFIA, 2019). In comparison, the average As level (0.94 mg/kg) in the current work was higher than the reported values for cultured and wild-caught Coho salmon (Luvonga et al., 2021). However, our mean value was below those found in dogfish (50 μg/g ww; North Sea, Brazil), catfish (8.9 μg/g ww; Atlantic Ocean, Brazil; Gao et al., 2018), flounder (3.3; USA; Burger and Gochfeld, 2005), fish from Sweden (1.28 mg/kg; Kollander et al., 2019), and shark (Cunningham et al., 2019). Additionally, the As average concentrations exhibited by two tuna species in this study were either below or the same as those in tuna samples from Belgium (1.43 mg/kg; Ruttens et al., 2012). Kruskal-Wallis test showed a statistical difference (p *<* 0.05) in As levels among pelagic fish (ahi tuna: Indonesia, vs. Atlantic salmon vs. tilapia; and Pacific whiting vs. pink salmon vs. sockeye salmon); and demersal fish (pollock vs. Pacific cod–USA and China). Tuna is particularly, a predator fish and potentially will accumulate As from the food web. Notwithstanding, there was no significant difference (*p* > 0.05) in As concentrations among pelagic fish (e.g., ahi tuna–Indonesia vs. ahi tuna–Vietnam; and Atlantic salmon: Chile vs. pink salmon–China); and benthic fish (pollock vs. Pacific cod–China and USA.

Cadmium is a nonessential element, classified as a human carcinogen, and toxicities can result in bone demineralization and renal dysfunction (EFSA, 2009a). Food is the major source of Cd exposure in the population (JECFA, 1989). About 0.4% of all samples exceeded the Cd limit (0.2 ppm). The average Cd concentration in the present work (Table 3) was highest in ahi tuna from Vietnam (0.079 mg/kg) and lowest in Ocean perch (0.018 mg/kg). The average concentrations found in the examined fish species were below the 0.2 mg/kg maximum allowable concentration (MAFF, 1998). Yet, the mean Cd level in tilapia (0.059 mg/kg) was slightly above the European Commission (EC) maximum permissible level (MPL; 0.05 mg/kg) for fish (Official Journal of the European Union, 2008, Official Journal of the European Union, 2014, Official Journal of the European Union, 2015). The number of samples (in parenthesis) in exceedance of the MPL was Pacific cod–USA (6), Pacific cod–China (1), sockeye salmon (6), tilapia (14), catfish (3), Atlantic salmon (1), and ahi tuna–Indonesia (1). The average Cd level (all species; this study) was not different from the concentrations found in demersal fish from Turkey (*Solea lascaris*: 0.04 mg/kg; Yilmaz et al., 2010), catfish (*Clarias fuscus*, China; Leung et al., 2014), and Atlantic cod (*Gadus morhua L.;* Poland; Polak-Juszczak and Podolska, 2021). However, our mean value was below the level in Gulf fish (0.53 mg/kg; Cunningham et al., 2019) but comparable to the levels attained in *Canthidermis sufflamen* and *Heteropriacanthus fulgens* (Lozano-Bilbao et al., 2021, Table 4). Kruskal -Wallis test results signaled that Cd levels differed (p *<* 0.05) between demersal and pelagic fish samples (e.g., flounder vs. tilapia; and tilapia vs. Pacific cod–USA vs. Pollock) and between farm-raised and wild-caught (e.g., Atlantic salmon: Chile vs. Ocean perch; catfish vs. Ocean perch; and catfish vs. tilapia), and among the sampled pelagic species (e.g., ahi tuna–Indonesia vs. ahi tuna -Vietnam; and Pacific whiting vs. tilapia). Nevertheless, there were no statistical differences (p > 0.05) in Cd concentrations among the epipelagic species (pink salmon–China, wild-caught vs. Atlantic salmon: Chile, farm-raised); and wild-caught benthic fish (Pacific cod–USA vs. Pacific cod–China). Additionally, homogeneity (p > 0.05) in Cd concentrations was observed among demersal (pollock vs. flounder vs. Pacific cod vs. catfish) and pelagic (Atlantic salmon vs. pink salmon vs. Pacific whiting vs. sockeye salmon) fish species.

Chromium, an essential element, can exist predominantly in two oxidation states (Cr^III^ and Cr^VI^). Cr (III) acts as a critical cofactor in insulin action and engages in carbohydrate, lipid, and protein metabolism. Cr ^III^ is a natural dietary constituent present in foods. Conversely, Cr^V^ is carcinogenic according to the International Agency for Research on Cancer (IARC) (WHO, 1980). Closely 58% of all samples exceeded the Cr limit (0.1 ppm). From Table 3, the highest average Cr concentration was exhibited by tilapia (0.21 mg/kg) and the lowest mean level was achieved in Ocean perch (0.09 mg/kg). The average Cr in this study was consistently lower than the values reported for three fishes from Turkey (*S. lascaris*: 0.70 mg/kg; *L. budegassa*: 0.32 mg/kg; and *T. lucerna*: 0.65 mg/kg; Yilmaz et al., 2010). Anyhow, our average Cr was greater than the Brazilian standard (0.1 mg Cr/kg) except for Ocean perch. Similarly, our average (all samples; 0.14 mg/kg; Table 3) was higher than the values (mg/kg) obtained for Baltic cod (0.03), Atlantic cod (0.03), and saithe (0.04) species (Polak-Juszczak and Podolska, 2021) but below the concentration in tub gurnard (*Triglia lucerne,* Turkey; Yilmaz et al., 2010). From the Kruskal-Wallis test results, differences (*p <* 0.05) were found between the concentrations of Cr among pelagic fish (ahi tuna–Indonesia vs. ahi tuna–Vietnam); and between demersal and pelagic fish samples (flounder vs. Ocean perch; Pollock vs. tilapia; catfish vs. tilapia; and Pacific cod–USA and China vs. Pacific whiting). Nonetheless, no significant difference (*p* > 0.05) was found in Cr concentrations among pelagic (Atlantic pink salmon, farm-raised vs. Alaskan sockeye salmon, wild; and Ocean perch vs. Pacific whiting) and demersal (e.g., catfish vs. flounder; catfish vs. Pacific cod–USA and China; and flounder vs. pollock) fish samples.

Copper is an essential micronutrient that participates in several enzyme processes, synthesis of connective tissues, and many other functions (EGV, 2003). Approximately 0% of all samples exceeded the Cu limit (20 ppm). The average Cu concentration was lowest in Ocean perch (0.14 mg/kg) and highest in pink salmon (0.42 mg/kg) (Table 3). Among the species examined, none of the samples was in exceedance of the 50 mg/kg (MAFF, 1998) or 30 mg/kg (FAO, 1983) safe limits for fish. Symptoms of acute copper poisoning include salivation, nausea, vomiting, epigastric pain, diarrhea, and renal failure (WHO, 1996). The mean Cu concentration (this study; 0.30 mg/kg) was lower than the concentrations in three demersal fish muscle samples from Turkey (*S. lascaris*: 5.64 mg/kg; *L. budegassa*: 6.24 mg/kg; and *T. lucerna*: 4.19 mg/kg; Yilmaz et al., 2010). The Cu average levels (mg/kg) attained in Pacific cod (USA) samples (0.17) and China (0.15) were lower or comparable to those for Baltic cod (0.22), Atlantic cod (0.24), and saithe (0.23) species (Polak-Juszczak and Podolska, 2021), and catfish (China; Leung et al., 2014). The Cu average for all samples (this study) was below the concentrations reported for three fish species (*Balistes capriscus*, *C. sufflamen*, and *H. fulgens;* Lozano-Bilbao et al., 2021). Results of the Kruskal-Wallis test suggested no significant differences (*p* > 0.05) between Cu concentrations in pelagic wild-caught fish (ahi tuna–Indonesia vs. ahi tuna–Vietnam; and the tuna species vs. Pacific whiting); farm-raised pelagic fish species (Atlantic salmon vs. tilapia), and demersal wild-caught fish (Pacific cod–USA and China vs. flounder vs. pollock). Contrarily, statistical differences (p *<* 0.05) were found between pelagic wild-caught fish (e.g., ahi tuna–Indonesia vs. Ocean perch; Pacific whiting vs. sockeye salmon; ahi tuna–Vietnam vs. Ocean perch; and pink vs. sockeye salmons). Further, non-homogeneity (p *<* 0.05) in metal concentrations was achieved between wild-caught and farm-raised species (e.g., ahi tuna–Vietnam vs. catfish; Ocean perch vs. tilapia; and sockeye salmon vs. catfish).

Nickel influences iron absorption and metabolism and may be an essential component of the hemopoietin process (EGV, 2003). In the present study, 0% of all samples exceeded the Ni limit (10 ppm). The maximum average Ni level (0.15 mg/kg) was observed in Pacific whiting while the minimal average level (0.07 mg/kg) was achieved in catfish. Ni concentrations among the analyzed species were below the Egyptian standard (10 mg/kg; EOS, 1993). Regardless, acute Ni exposure is associated with gastrointestinal problems while chronic inhalation can result in increased lung cancer risk (EFSA, 2015, EGV, 2003). The Ni average values were consistent with published values in pelagic fish (Blue whiting, and European hake) but lower in Atlantic bluefin tuna and swordfish (Storelli et al., 2020). The Kruskal-Wallis analysis results pinpointed that Ni concentrations were significantly different (p *<* 0.05) between pelagic fish (pink salmon vs. sockeye salmon vs. tilapia; Pacific whiting vs. tilapia; and Ocean perch vs. tilapia) and demersal fish (catfish vs. Pacific cod–USA vs. ahi tuna–Vietnam). On the contrary, there was no statistical difference (p > 0.05) in Ni concentrations among pelagic (tuna species vs. tilapia; Atlantic salmon vs. pink salmon); and demersal (flounder vs. Pacific cod–USA and China vs. pollock) fish. The Ni value for all samples in this study (0.09 mg/kg) was comparable to the concentration found in *B. capriscus* (Lozano-Bilbao et al., 2021).

Lead is a class 2B carcinogen, which causes sterility, neonatal mortality, morbidity, and mental retardation in children (WHO, 1996). Acute Pb exposure can induce appetite loss, headaches, hypertension, stomach discomfort, renal dysfunction, fatigue, and insomnia (ATSDR, 2007). About 0.8% of all samples exceeded the Pb limit (0.5 ppm). The average concentrations of Pb in the present work ranged from 0.13 mg/kg to 0.22 mg/kg among the sampled species. The highest mean Pb level was observed in Pollock and the lowest average was in Ocean perch (Table 3). Of all samples, the mean concentration of Pb (0.17 mg/kg) in this study was below the MAC (2.0 mg/kg; MAFF, 1998; Official Journal of the European Union, 2008). However, the European Union action limit (0.30 mg/kg) was exceeded in 8% of samples including pollock with a maximal value (0.82 mg/kg). Our Pb average level (0.17 mg/kg) was lower than those found in *B. capriscus*, *C. sufflamen*, and *H. fulgens* (Lozano-Bilbao et al., 2021). The Kruskal-Wallis test confirmed no statistically significant (p > 0.05) differences in Pb concentrations among the investigated species and our values were consistent with the insignificant Pb levels found in fishes from the Adriatic Sea (Bilandžić et al., 2011) and Bahía Blanca (Argentina; La Colla et al., 2017).

Zinc is an essential element linked to many metalloenzyme processes, synthesis of genetic material, and degradation of proteins, lipids, and carbohydrates (EGV, 2003). Around 0% of all samples exceeded the Zn limit (30 ppm). The average Zn concentrations (Table 4) across the species were comparable (range: 2.34–3.70 mg/kg ww). Maximal Zn values (mg/kg) were observed in catfish (6.18), ahi tuna–Vietnam (6.16), and flounder (5.65) samples. The highest average Zn concentration (3.70 mg/kg ww) was found in flounder, while the lowest mean was observed in Pacific cod–USA (2.34 mg/kg). Nonetheless, Zn toxicity includes anemia, and increased plasma cholesterol among others (EGV, 2003). Irrespective of the species studied, the average Zn concentration (2.83 mg/kg; all samples) in the present study (Table 3) was lower than the permitted amount (50 mg/kg; MAFF, 1998), and the 30 mg/kg allowable limit in fish (FAO, 1983). In comparison with other previous works, the mean Zn level was below the values reported for three demersal fish species from Turkey (*Triglia lucerna*: 28.2 mg/kg, *Lophius budegassa*: 20.8 mg/kg; and *Solea lascaris*: 27.5 mg/kg; Yilmaz et al., 2010). Similarly, the average Zn level in Pacific cod–China (2.15 mg/kg ww) was below the reported value for Atlantic cod (3.6 mg/kg ww; Polak-Juszczak and Podolska, 2021). Our Zn average for all samples (2.83 mg/kg) was below the values reported for *B. capriscus*, *C. sufflamen*, and *H. fulgens* (Table 4). Kruskal-Wallis test revealed insignificant statistical differences (p > 0.05) between pelagic (Ocean perch vs. tilapia vs. Pacific whiting; Atlantic salmon vs. pink salmon vs. sockeye salmon) and demersal (catfish vs. pollock; Pacific cod–USA and China vs. Pollock) fish samples. All the same, non-homogeneity (p *<* 0.05) in Zn concentrations was observed between pelagic (Ocean perch vs. pink salmon) and demersal (flounder vs. pollock vs. pacific cod–China and USA; catfish vs. flounder) fish. A similar difference was reported among bottom-dwelling and pelagic species (Yilmaz et al., 2010).

Mercury arises from natural and anthropogenic sources (e.g., volcanoes, gold mining, chloralkali production, batteries) and cycles between the ocean, land, and atmosphere. Mercury occurs as (i) elemental or metallic mercury (Hg^o^), (ii) inorganic mercury [mercurous (Hg_2_^2+^) and mercuric (Hg^2+^) cations], and (iii) organic mercury. CH_3_Hg is the usual form in foods (EFSA, 2012). Hg is neurotoxic (ATSDR, 2007) and exposure can lead to tremors, vomiting, fatigue, etc. Codex Alimentarius and MAFF guideline for fish is set at 0.05 mg/kg except in predatory fish (e.g., tuna, shark) with a maximum limit of 1.0 mg/kg. Approximately 0% of all samples exceeded the Hg limit (0.5 ppm). The average THg concentrations across the fish species (this study) ranged from 0.001 mg/kg (tilapia) to 0.10 mg/kg (ahi tuna–Indonesia). For all samples, the average and maximum THg concentrations were 0.04 mg/kg and 0.27 mg/kg, respectively (Table 3). Regardless of the species studied, the average THg level (0.036 mg/kg) was below the prescribed limit (0.5 mg/kg; MAFF, 1998) and comparable to the Hg concentration (0.106 μg/g) in fish from Malaysia (Anual et al., 2018). The mean THg concentrations in red piranha (*Pygocentrus natterer*; wild-caught; 0.56 mg/kg; Custódio et al., 2020), and tuna samples (0.22 mg/kg) were higher than the average value for ahi tuna samples from Indonesia (0.05 mg/kg; this study). Similarly, the mean THg level (catfish—farmed: 0.003 mg/kg) was lower than the mean concentration in catfish from the wild (0.07 mg/kg; Custódio et al., 2020). Yet, the average THg level in farmed tilapia (this study; 0.001 mg/kg) was lower than the concentrations in farm-raised Nile tilapia from Brazil (0.02 mg/kg; Custódio et al., 2020) and pink salmon (0.0419 mg/kg; Burger et al., 2014). In the present work, Hg concentrations differed significantly (p < 0.05) between pelagic and benthic (Ahi tuna–Indonesia and Vietnam vs. catfish vs. pollock vs. Pacific cod) fish and among pelagic (Ahi tuna–Indonesia and Vietnam vs. pink salmon vs. sockeye salmon; ocean perch vs. tilapia) and benthic (flounder vs. pollock) fish samples.

### 3.3 Comparison of metal concentrations in wild vs. farmed fishes

Kruskal-Wallis test revealed significant differences in As and Hg median concentrations between farm-raised and wild fish. A significant difference (p < 0.05) in median As concentrations was observed between wild (0.61 mg/kg; n = 180) and farmed (0.041 mg/kg; n = 59) fish. Similarly, there was a statistical difference (p < 0.05) in the median concentrations (mg/kg) of Hg between farmed (0.003) and wild (0.047) fish. In this instance, fish from the wild may be exposed to more contaminants through diet and habitat. Median levels of Zn, Pb, Ni, and Cu across farmed and wild fish were homogeneous (p > 0.05). Moreover, Zn levels (mg/kg) in wild (average: 2.84; median: 2.73) and farmed (average: 2.80; median: 2.75) fish were comparable and statistically insignificant (*p* > 0.05). Likewise, median Pb values (0.16 mg/kg) were the same in the groups of fish. Nevertheless, noticeable differences were found in Cd (*p-value* = 0.00130078) and Cr (*p-value* = 0.0106512) median levels of farmed vs. wild fish. In comparison, a significant difference was attained in Cu levels across all sites in at least one wild fish species and farmed fish from the Mediterranean (Kalantz et al., 2013). Furthermore, under at least one anoxic site, at least one farmed fish species, had significantly higher levels of As, Hg, Cd, and Ni in muscle in comparison to those of wild fish species (Kalantz et al., 2013).

### 3.4 Spearman's rank correlation

**Figure 1:**
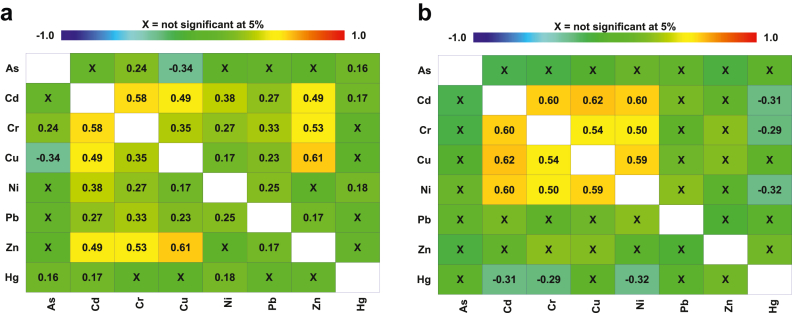
Spearman's rank coefficients (r) for correlations of metals/metalloid levels among (a) wild, (b) farmed fish species from the Missouri market. The coefficients indicated are statistically significant at *p* < 0.05.

Figure 1a presents Spearman's rank correlation coefficients (p < 0.05) of trace elements among wild-caught fish species. Significant correlations (p < 0.05) were observed between some trace elements. Moderate to strong associations (r^2^: 0.49–0.61; p < 0.05) was achieved for Cr—Cd; Cu—Cd, Zn—Cd, and Zn—Cr, with Zn—Cu the highest (r^2^: 0.61). Pb correlated significantly (p < 0.05) but weakly (r^2^: 0.17–0.33) with Zn, Ni, Cu, Cr, and Cd. Weak correlations (r^2^: 0.16–0.18) were attained for Hg vs. Ni, Hg vs. Cd, and Hg vs. As. Furthermore, As was weakly associated with Cr but negatively with Cu.

Figure 1b presents Spearman's rank correlation coefficients (p < 0.05) of trace elements among farmed fish species with moderate to strong relationships in Cr/Cu (r^2^: 0.54), Cr/Ni (r^2^: 0.50), and Ni/Cu (r^2^: 0.59). Zn, As, and Pb did not significantly (p > 0.05) correlate with other analyzed elements whereas Hg was significantly (p < 0.05) but negatively associated with Cd, Cr, and Ni (r^2^: −0.29 to −0.32). Ni, Cr, and Cu associations were similar in wild and farmed fish (Alam et al., 2002). The interrelationships of the elements may indicate common origins (e.g., food sources) or involvements in biochemical processes. Also, competition and homeostasis may play a role in the accumulation of metals in fish.

### 3.5 Human health evaluation

#### 3.5.1 Daily/weekly intakes of trace elements through fish muscle consumption

Table 5 summarizes the dietary intake (EDI: μg/kg body weight per day; and EWI: μg/kg body weight per week) values via fish muscle consumption in adults. The EWI values for Cd, Cr, Cu, Ni, Pb, Hg, and Zn were consistently lower than the respective PTWI value, which indicates no major health hazard to adult consumers.

**Table 5:** Mean dietary intake (EDI^a^; μg/kg body weight per day); and EWI^b^ (in parenthesis; μg/kg body weight per week assuming 70 kg body weight) of trace elements through fish muscle consumption in the adult population and regulatory values for metals in fish.

Fish species and number of samples	Origin	As	Cd	Cr	Cu	Ni	Pb	Zn	Hg
Sockeye salmon (n = 20)	USA	0.06 (0.44)	0.02 (0.12)	0.06 (0.43)	0.19 (1.31)	0.07 (0.46)	0.09 (0.60)	1.34 (9.40)	0.02 (0.14)
Atlantic salmon (n = 12)	Chile	0.02 (0.15)	0.01 (0.08)	0.06 (0.43)	0.11 (0.79)	0.04 (0.29)	0.07 (0.46)	1.34 (0.94)	0.002 (0.02)
Pink salmon (n = 20)	China	0.09 (0.6)	0.01 (0.07)	0.06 (0.45)	0.19 (1.36)	0.03 (0.21)	0.07 (0.50)	1.51 (10.5)	0.009 (0.062)
Ahi tuna (n = 12)	Vietnam	0.67 (4.67)	0.04 (0.26)	0.08 (0.57)	0.15 (1.07)	0.06 (0.39)	0.10 (0.68)	1.69 (11.8)	0.03 (0.24)
Ahi tuna (n = 20)	Indonesia	0.25 (1.74)	0.01 (0.08)	0.05 (0.37)	0.13 (0.89)	0.05 (0.32)	0.09 (0.61)	1.35 (0.95)	0.05 (0.33)
Tilapia (n = 22)	China	0.03 (0.18)	0.03 (0.19)	0.1 (0.68)	0.13 (0.88)	0.06 (0.42)	0.08 (0.58)	1.32 (9.24)	0.0005 (0.004)
Pollock (n = 15)	USA	0.57 (3.97)	0.01 (0.06)	0.05 (0.34)	0.41 (2.86)	0.03 (0.24)	0.10 (0.7)	1.18 (0.83)	0.004 (0.03)
Catfish (n = 25)	USA	0.04 (0.26)	0.01 (0.09)	0.05 (0.37)	0.08 (0.55)	0.03 (0.23)	0.07 (0.51)	1.27 (0.89)	0.001 (0.009)
Ocean perch (n = 18)	USA	0.22 (1.53)	0.01 (0.06)	0.04 (0.28)	0.07 (0.46)	0.04 (0.31)	0.06 (0.44)	1.11 (7.74)	0.02 (0.13)
Flounder (n = 16)	China	0.99 (0.7)	0.01 (0.07)	0.07 (0.05)	0.11 (0.78)	0.03 (0.23)	0.08 (0.54)	1.73 (12.1)	0.02 (0.13)
Pacific cod (n = 18)	China	1.0 (7.0)	0.01 (0.08)	0.08 (0.56)	0.07 (0.49)	0.04 (0.3)	0.08 (0.55)	1.19 (8.34)	0.02 (0.13)
Pacific cod (n = 21)	USA	1.83 (12.8)	0.02 (0.12)	0.05 (0.36)	0.08 (0.55)	0.05 (0.34)	0.07 (0.52)	1.09 (7.61)	0.03 (0.20)
Pacific whiting (n = 20)	USA	0.08 (0.59)	0.01 (0.7)	0.07 (0.47)	0.14 (0.98)	0.07 (0.5)	0.08 (0.56)	1.20 (0.84)	0.02 (0.16)
**PTWI**^c^		0.3^d^	2.5^e^	300^f^	3500^g^	35^h^	25^g^	7000^g^	4^i^

a EDI (estimated daily intake; μg/kg body weight per day).

b EWI (estimated weekly intake; μg/kg body weight per week).

c PTWI (provisional tolerable weekly intake; μg/kg body weight per week).

d Chronic-duration oral exposure (≥1 year) minimal risk level (MRL) for inorganic arsenic (iAs; As^III^ and As^V^); ATSDR, 2007.

e EFSA (European Food Safety Authority), 2009b.

f Tolerable daily Intake for Cr^III^; EFSA (European Food Safety Authority), 2014b.

g JECFA (Joint FAO/WHO Expert Committee on Food Additives), 2000.

h WHO (World Health Organization), 1993.

i EFSA (European Food Safety Authority), 2012.

The PTWI for Cd is 7.0 μg/kg body weight per week but at the seventy-third meeting of JECFA, the Committee rE-evaluated Cd in foods and established a provisional tolerable monthly intake (PTMI) of 25 μg/kg body weight per month, reflecting the long half-life of cadmium in humans (JECFA, 2013). The monthly average intake of Cd (range: 0.24–2.08 μg/kg body weight per month; Table 5) from the fish species was below the PTMI value.

The metalloid, arsenic (As) exhibited the highest percentage contributions (EWI/PTWI ratio) in Pacific cod–USA (85%), Pacific cod–China (47%), ahi tuna–Vietnam (31%), pollock (26%), ahi tuna–Indonesia (12%), and Ocean perch (10%). Additionally, the average EWI (Table 5) values of As in sockeye and pink salmons, tuna species, pollock, Ocean perch, flounder, Pacific cod, and Pacific whiting exceeded the PTWI for iAs (0.3 μg/kg body weight per week). According to the EFSA, fish and other seafood have a high total arsenic content (often in the range of 2–60 mg As/kg dry mass) but the concentrations of iAs are typically <0.2 mg As/kg dry mass. Consequently, the iAs content of samples in this study should be lower than the corresponding total As concentration. Concerning As, the consumption of demersal fish may pose risk to consumers, especially at high ingestion rates.

The EWI (μg/kg body weight per week) values of Hg ranged from 0.004 in tilapia to 0.33 in ahi tuna from Indonesia, which signified less hazard to consumers.

#### 3.5.2 Non-carcinogenic risk assessment

**Figure 2:**
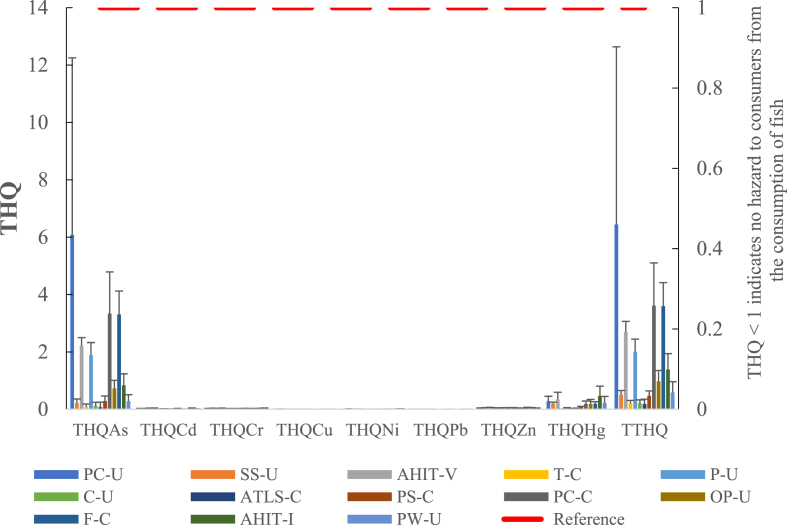
Non-carcinogenic risk (THQ: target hazard quotient) and total hazard index (TTHQ:total target hazard quotient) through the consumption of fish species from the Missouri market (ingestion rate of 32.5714 g per person per day). PC-U: Pacific cod (USA); SS-U: Sockeye salmon (USA); AHIT-V: AHI tuna (Vietnam); T-C: Tilapia (China); P-U: Pollock (USA); C-U: Catfish (USA); ATLS-C: Atlantic salmon (China); PS-C: Pink salmon (China); PC-C: Pacific cod (China); OP-U: Ocean perch (USA); F-C: Olive flounder (China); AHIT-I: AHI tuna (Indonesia); and PW-U: Pacific whiting (USA).

Figure 2 and Table 6 present the individual THQs for the trace elements and the cumulative risk (TTHQ) across the species. The non-cancer risk (THQ) values for the trace elements except arsenic were less than 1 (ranges: THQ_As_ 0.07–6.1; THQ_Cd_ 0.01–0.04; THQ_Cr_ 0.01–0.03; THQ_Cu_ 0.002–0.005; THQ_Ni_ 0.003–0.006; THQ_Pb_ 0.002–0.003; and THQ_Zn_ 0.04–0.06; and THQ_Hg_ 0.005–0.47). The average THQ_As_ for all species in this study was 1.45. Thus, the non-cancer risk values were within the acceptable limit for analyzed trace elements except arsenic. Nevertheless, arsenic levels in fish muscle posed the most non-cancer risk with the overall contribution in the range from 41% to 94% across the species. Pacific cod, flounder, and Pollock had the highest As contributions (92%–94%) while salmon, tilapia, and Pacific whiting achieved the lowest THQs (Figure 2; Table 6). Regarding the TTHQ_As_ of the analyzed samples, the exceedances (%) relative to the benchmark (10^−5^) in samples (with the average THQ_As_ in parenthesis) were as follows: ahi tuna–Vietnam 100% (2.7); ahi tuna–Indonesia 25% (1.4); flounder: 100% (3.6); Pacific cod–China 100% (3.6); Pacific cod–USA 100% (6.4); and pollock 100% (2.0) and Ocean perch 6% (1.0). Nonetheless, the TTHQ values (range: 0.2–6.4) for the eight trace elements through fish muscle consumption, in some instances, were greater than the threshold (i.e., TTHQ >1), which suggested potential adverse effects in the adult group. The analyzed fish species such as flounder, Pacific cod, tuna, and pollock samples posed more potential risks than other species from their TTHQ values (Figure 2 and Table 6). Nonetheless, the potential risk from intake of As from fish muscle may be insignificant assuming that <10% of As (total) in samples were in the inorganic form (iAs; toxic form).

**Table 6:** Toxic hazard quotients (THQ) and total target hazard quotient (TTHQ) for trace elements in commercial fish species from the Missouri market.

Fish species	Origin	Statistic	THQ_As_	THQ_Cd_	THQ_Cr_	THQ_Cu_	THQ_Ni_	THQ_Pb_	THQ_Zn_	THQ_Hg_	TTHQ	Threshold
Pacific Cod	USA	Average	6.08	0.017	0.017	0.002	0.004	0.002	0.036	0.28	6.44	1.0
Pacific Cod	USA	SD	6.17	0.014	0.012	0.001	0.002	0.001	0.014	0.17	6.2	
Sockeye Salmon	USA	Average	0.21	0.018	0.02	0.005	0.006	0.002	0.045	0.2	0.5	1.0
Sockeye Salmon	USA	SD	0.15	0.013	0.019	0.002	0.005	0.001	0.013	0.05	0.15	
AHI Tuna	Vietnam	Average	2.22	0.037	0.027	0.004	0.005	0.003	0.056	0.34	2.7	1.0
AHI Tuna	Vietnam	SD	0.28	0.005	0.004	0.001	0.001	0.001	0.013	0.25	0.37	
Tilapia	China	Average	0.08	0.03	0.032	0.003	0.005	0.002	0.044	0.01	0.21	1.0
Tilapia	China	SD	0.1	0.016	0.01	0.001	0.002	0.001	0.009	0	0.1	
Pollock	USA	Average	1.89	0.009	0.016	0.003	0.003	0.003	0.039	0.04	2	1.0
Pollock	USA	SD	0.44	0	0.006	0.002	0.002	0.002	0.009	0.01	0.44	
Catfish	USA	Average	0.13	0.012	0.018	0.002	0.003	0.002	0.042	0.01	0.22	1.0
Catfish	USA	SD	0.12	0.008	0.009	0.001	0.004	0.001	0.016	0.01	0.11	
ATL Salmon	Chile	Average	0.07	0.012	0.021	0.003	0.004	0.002	0.045	0.02	0.18	1.0
ATL Salmon	Chile	SD	0.08	0.005	0.013	0.001	0.003	0.002	0.005	0.01	0.1	
Pink Salmon	China	Average	0.29	0.01	0.022	0.005	0.003	0.002	0.05	0.09	0.47	1.0
Pink Salmon	China	SD	0.18	0.002	0.006	0.002	0.002	0.001	0.01	0.02	0.17	
Pacific Cod	China	Average	3.34	0.015	0.027	0.002	0.004	0.002	0.04	0.19	3.61	1.0
Pacific Cod	China	SD	1.45	0.017	0.007	0.001	0.002	0.001	0.008	0.1	1.49	
Ocean Perch	USA	Average	0.73	0.008	0.014	0.002	0.004	0.002	0.037	0.18	0.98	1.0
Ocean Perch	USA	SD	0.28	0.001	0.013	0.001	0.005	0.001	0.004	0.16	0.38	
Flounder	China	Average	3.31	0.009	0.024	0.003	0.003	0.002	0.057	0.19	3.6	1.0
Flounder	China	SD	0.81	0.001	0.008	0.001	0.002	0.001	0.011	0.08	0.82	
AHI Tuna	Indonesia	Average	0.83	0.017	0.018	0.003	0.004	0.002	0.045	0.47	1.39	1.0
AHI Tuna	Indonesia	SD	0.41	0.027	0.016	0.001	0.004	0.002	0.013	0.34	0.55	
Pacific Whiting	USA	Average	0.28	0.009	0.022	0.004	0.006	0.002	0.04	0.22	0.59	1.0
Pacific Whiting	USA	SD	0.23	0.001	0.025	0.002	0.011	0.001	0.005	0.23	0.38	

THQ_Hg_ (all samples; 6.6) reported for Atlantic bluefin tuna (Milatou et al., 2020) were higher than the observed values for the fish species (this study). THQ values of Pb, Cd, Hg, and As for Cod were below one (Polak-Juszczak and Podolska, 2021) but THQ_Pb_ was greater than one for *Pampus argenteus* and *Tenualosa ilisha* from some coastal areas (Bristy et al., 2021).

#### 3.5.3 Carcinogenic risk assessment

**Figure 3:**
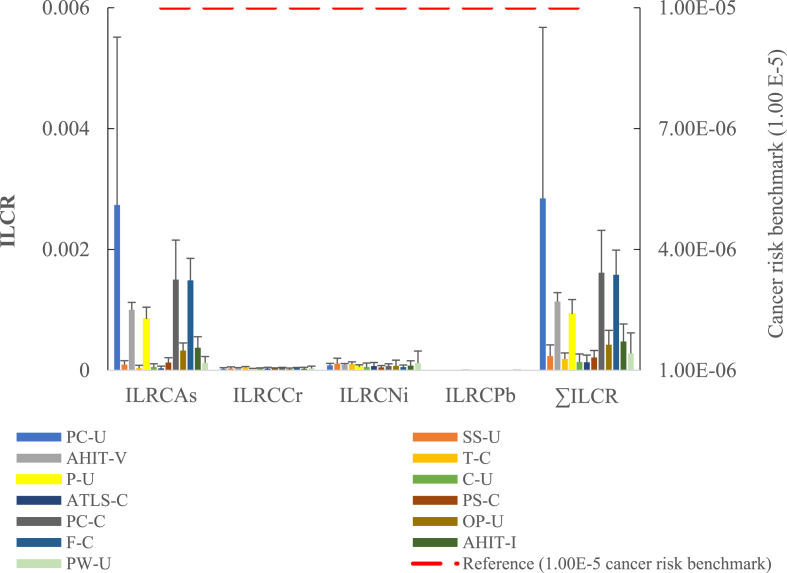
Incremental lifetime cancer risk (ILCR) and the sum of the ILCR (∑ILCR) via the consumption (ingestion rate of 32.5714 g per person per day) of fish species from the Missouri market. PC-U: Pacific cod (USA); SS-U: Sockeye salmon (USA); AHIT-V: AHI tuna (Vietnam); T-C: Tilapia (China); P-U: Pollock (USA); C-U: Catfish (USA); ATLS-C: Atlantic salmon (China); PS-C: Pink salmon (China); PC-C: Pacific cod (China); OP-U: Ocean perch (USA); F-C: Olive flounder (China); AHIT-I: AHI tuna (Indonesia); and PW-U: Pacific whiting (USA).

Figure 3 and Table 7 summarize the ILCR and the ∑ILCR values for exposure to trace elements from fish muscle. Among all the species, the average ILCR for As, Cr, Ni, and Pb were 6.5 × 10^−4^; 3.2 × 10^−5^; 7.9 × 10^−5^; and 6.7 × 10^−7^, respectively. The calculated average ILRC risk values (Figure 3 and Table 7) for As, Cr, and Ni and their sum (∑ILCR) exceeded the benchmark (1.0 × 10^−5^), which revealed potential cancer risk from fish muscle consumption. Nevertheless, the ILCR_Pb_ values across the species presented an insignificant cancer risk to the adult population. In comparison, the cancer risk from As in Atlantic cod (Polak-Juszczak and Podolska, 2021) and Cd in *P. argenteus, Sardinella longiceps,* and *T. ilisha* fish species (Bristy et al., 2021) exceeded the cancer benchmark(s) but insignificant for Pb in samples (Bristy et al., 2021).

**Table 7:** Estimation of incremental lifetime risk (ILCR) and the sum of the cancer risks for the trace elements (As, Ni, Cr, and Pb) through fish consumption in the adult risk group.

Statistics	Fish species	Country	ILRC_As_	ILRC_Cr_	ILRC_Ni_	ILRC_Pb_	∑ILCR	Benchmark applied
Average	Pacific Cod	USA	2.7E − 03	2.6E − 05	8.2E − 05	6.3E − 07	2.8E − 03	1.00E − 5
SD	Pacific Cod	USA	2.8E − 03	1.8E − 05	3.4E − 05	3.5E − 07	2.8E − 03	
Average	Sockeye Salmon	USA	9.5E − 05	3.1E − 05	1.1E − 04	7.2E − 07	2.4E − 04	1.00E − 5
SD	Sockeye Salmon	USA	6.6E − 05	2.8E − 05	9.0E − 05	3.5E − 07	1.4E − 04	
Average	AHI Tuna	Vietnam	1.0E − 03	4.1E − 05	9.6E − 05	8.2E − 07	1.1E − 03	1.00E − 5
SD	AHI Tuna	Vietnam	1.2E − 04	5.9E − 06	1.8E − 05	2.5E − 07	1.3E − 04	
Average	Tilapia	China	3.8E − 05	4.8E − 05	1.0E − 04	7.1E − 07	1.9E − 04	1.00E − 5
SD	Tilapia	China	4.6E − 05	1.5E − 05	3.8E − 05	3.0E − 07	6.6E − 05	
Average	Pollock	USA	8.5E − 04	2.4E − 05	5.7E − 05	8.5E − 07	9.3E − 04	1.00E − 5
SD	Pollock	USA	2.0E − 04	8.6E − 06	3.4E − 05	6.8E − 07	1.9E − 04	
Average	Catfish	USA	5.7E − 05	2.7E − 05	5.5E − 05	6.2E − 07	1.4E − 04	1.00E − 5
SD	Catfish	USA	5.3E − 05	1.3E − 05	6.6E − 05	2.8E − 07	9.9E − 05	
Average	ATL Salmon	Chile	3.3E − 05	3.1E − 05	7.0E − 05	5.6E − 07	1.3E − 04	1.00E − 5
SD	ATL Salmon	Chile	3.8E − 05	2.0E − 05	6.0E − 05	5.1E − 07	8.9E − 05	
Average	Pink Salmon	China	1.3E − 04	3.2E − 05	5.0E − 05	6.0E − 07	2.1E − 04	1.00E − 5
SD	Pink Salmon	China	7.9E − 05	8.6E − 06	2.9E − 05	3.0E − 07	7.9E − 05	
Average	Pacific Cod	China	1.5E − 03	4.0E − 05	7.3E − 05	6.7E − 07	1.6E − 03	1.00E − 5
SD	Pacific Cod	China	6.5E − 04	1.0E − 05	3.7E − 05	3.9E − 07	6.6E − 04	
Average	Ocean Perch	USA	3.3E − 04	2.0E − 05	7.5E − 05	5.3E − 07	4.2E − 04	1.00E − 5
SD	Ocean Perch	USA	1.3E − 04	1.9E − 05	9.3E − 05	3.4E − 07	1.4E − 04	
Average	Flounder	China	1.5E − 03	3.6E − 05	5.5E − 05	6.6E − 07	1.6E − 03	1.00E − 5
SD	Flounder	China	3.6E − 04	1.2E − 05	3.3E − 05	2.6E − 07	3.6E − 04	
Average	AHI Tuna	Indonesia	3.7E − 04	2.7E − 05	7.8E − 05	7.4E − 07	4.8E − 04	1.00E − 5
SD	AHI Tuna	Indonesia	1.8E − 04	2.4E − 05	8.0E − 05	5.9E − 07	2.1E − 04	
Average	Pacific Whiting	USA	1.3E − 04	3.3E − 05	1.2E − 04	6.8E − 07	2.8E − 04	1.00E − 5
SD	Pacific Whiting	USA	1.0E − 04	3.7E − 05	2.0E − 04	3.0E − 07	2.5E − 04	

## 4 Conclusion

The average metals/metalloid concentrations found in eleven commercial fish species from the Missouri market followed the order Zn > As > Cu > Pb > Cr > Ni > Hg > Cd. Among the essential elements, Zn and Cu were the most abundant in muscle. About the PTEs, arsenic followed by Pb was the most accumulated element across the species. The concentrations of As in Pacific cod and tuna species gave the highest percentage contributions relative to other analyzed elements. Of all the samples analyzed, the exceedances (in parenthesis) of the maximum limits were Cd (0.4%), As (4.2%), Cr (58%), Cu (0%), Ni (0%), Pb (0.8%), Zn (0%), and THg (0%). Only As found in certain pelagic (ahi tuna–Vietnam) and demersal (pollock, flounder, and Pacific cod) samples exceeded the weekly intake limit. Thus, most analyzed samples indicated less health concern. The non-parametric Kruskal-Wallis test results showed statistically significant differences (p *<* 0.05) in Cr, Ni, Cd, As, and Hg levels in some analyzed demersal and pelagic fish samples. Also, Kruskal-Wallis test results proved that median Hg and As concentrations differed significantly (p *<* 0.05) between farmed and wild fish, with higher values observed in wild fish. Nonetheless, median levels of Zn, Pb, Ni, and Cu between farmed and wild fish were not statistically significant (p > 0.05).

Across the species, Cd, Cr, Cu, Ni, Pb, and Zn posed the least non-cancer risk followed by As and Hg. However, THQ_As_ values were highest (i.e., THQ >1; 6%–100% exceedances in samples) in some demersal (e.g., Pacific cod, pollock, and flounder) and pelagic (e.g., tuna) fish samples, which pinpointed potential adverse effects.

Concerning cancer risk, the calculated ILCR and ∑ILCR values from exposure to Ni, As, and Cr exceeded the benchmark (10^−5^), which illustrated potential carcinogenesis among adult consumers. Despite the potential risk of heavy metals through fish consumption, fish remains an important source of essential micronutrients, proteins, EPA, and DHA for adequate human nutrition.

We recommend frequent exposure risk evaluation of commercial fish species (cultured and wild fish) to protect public health.

## Declarations

### Author contribution statement

Abua Ikem: Conceived and designed the experiments; Performed the experiments; Analyzed and interpreted the data; Contributed reagents, materials, analysis tools or data; Wrote the paper.

Jimmie Garth: Analyzed and interpreted the data; Contributed reagents, materials, analysis tools or data.

### Funding statement

Dr. Abua Ikem was supported by National Institute of Food and Agriculture [2015-38821-24386].

### Data availability statement

Data included in article/supp. material/referenced in article.

### Declaration of interests statement

The authors declare no conflict of interest.

### Additional information

No additional information is available for this paper.
